# Clinical and bacteriological outcomes in patients with urinary tract infections presenting to primary care in Harare, Zimbabwe: a cohort study

**DOI:** 10.12688/wellcomeopenres.16789.1

**Published:** 2021-06-01

**Authors:** Ioana D. Olaru, Mutsawashe Chisenga, Shunmay Yeung, Prosper Chonzi, Kudzai P.E. Masunda, Rashida A. Ferrand, Katharina Kranzer

**Affiliations:** 1 London School of Hygiene & Tropical Medicine, London, WC1E 7HT, UK; 2 Biomedical Research and Training Institute, 10 Seagrave Road, Avondale, Harare, Zimbabwe; 3 Department of Paediatric Infectious Disease, St Mary’s Imperial College Hospital, Praed St, Paddington, London, W2 1NY, UK; 4 Department of Health, Harare City Council, Rowan Martin Building, 1 Pennefather Avenue, Harare, Zimbabwe; 5 Division of Infectious and Tropical Medicine, Medical Centre of the University of Munich, Leopoldstrasse, Munich, 80802, Germany

**Keywords:** AMR, antibiotic resistance, cystitis, UTI

## Abstract

**Background**: Treatment for urinary tract infections (UTIs) is usually empiric and is based on local antimicrobial resistance data. These data, however, are scarce in low-resource settings. The aim of this study is to determine the impact of antibiotic treatment on clinical and bacteriological outcomes in patients presenting with UTI symptoms to primary care in Harare.

**Methods**: This cohort study enrolled participants presenting with UTI symptoms to 10 primary healthcare clinics in Harare between July 2019 and July 2020. A questionnaire was administered and a urine sample was collected for culture. If the urine culture showed growth of ≥10 ^5 ^colony forming units/mL of a uropathogen, a follow up visit at 7-21 days was conducted.

**Results**: The analysis included 168 participants with a median age of 33.6 years (IQR 25.1-51.4) and of whom 131/168 (78.0%) were female. Effective treatment was taken by 54/168 (32.1%) participants. The urine culture was negative at follow up in 41/54 (75.9%) of participants who took appropriate treatment and in 33/114 (28.9%, p<0.001) of those who did not. Symptoms had improved or resolved in 52/54 (96.3%) of those on appropriate treatment and in 71/114 (62.3%, p<0.001) of those without.

**Conclusion**: The findings of this study show that effective treatment leads to symptom resolution and bacterial clearance in people presenting with UTIs to primary care. Although UTIs are not life-threatening and can resolve without treatment, they do impact on quality of life, highlighting the need for optimised treatment recommendations.

## Introduction

Urinary tract infections (UTIs) are very common in women, with half of women reporting having had at least one episode by 32 years of age ^1^, while they are rare in men under the age of 60 years ^2^. Many women with a UTI experience moderate to severe symptoms impacting their daily life ^1^. Antibiotic treatment is mainly empiric and management recommendations are usually informed by local antimicrobial resistance (AMR) data collected as part of continuous surveillance ^3^. Such data are not widely available in many countries in sub-Saharan Africa ^4^. As a result, treatment recommendations are usually not informed by local or regional data, which may result in patients receiving ineffective empiric treatment. The aim of this study is to determine the impact of antibiotic treatment on clinical and bacteriological outcomes in patients presenting with UTI symptoms to primary care in Harare.

## Methods

The data were collected as part of the Antimicrobial Resistance in Gram-negative bacteria from Urinary Specimens (ARGUS) study, which enrolled patients presenting with UTI symptoms to ten primary healthcare clinics in Harare between July 2019 and July 2020. Details of the ARGUS study have been described elsewhere ^5^. Briefly, adult patients (≥18 years) reporting the current presence of at least two symptoms suggestive of a UTI and who had not been recently discharged from hospital (within the previous 72 hours) and did not have an indwelling urinary catheter were eligible for inclusion into the study. Eligible patients presenting to the participating clinics were consecutively recruited into the study. After provision of informed consent, a questionnaire on demographics, clinical history and treatment was administered and a urine sample collected for culture. Bacterial culture, identification and antimicrobial susceptibility testing (AST) were performed using conventional microbiology techniques. AST was interpreted using the EUCAST standards ^6^. ATCC reference isolates were used to ensure the quality of bacterial identification and AST.

If the urine culture showed growth of ≥10 ^5^ colony forming units/mL of a uropathogen, a follow up visit at 7–21 days was conducted when another urine sample was collected and a questionnaire on symptoms, treatment and healthcare seeking was administered. Bacteriological cure was defined as a negative urine culture on follow up. A favourable clinical outcome was defined as the resolution or improvement of symptoms at follow up.

Treatment was prescribed by the clinic nurses according to routine practice and followed the national guidelines, which recommend amoxicillin or fluoroquinolones as first-line treatment for UTIs ^7^. Participants were considered to have received effective treatment if the antibiotic prescribed was shown to have activity against the pathogen as per the AST result and the participant reported having taken the antibiotic. Participants who had positive cultures on follow up and were symptomatic were prescribed effective treatment according to AST. Pregnant women were treated irrespective of symptoms.

This analysis excluded participants in whom the follow-up visit was conducted late (>21 days post enrolment), if the treatment prescribed was not recorded, and if the sample could not be processed. Statistical analyses were performed in STATA v.15 (StataCorp, TX, USA). Univariable analyses were performed using the χ square test for categorical variables and Mann Whitney U test for continuous variables. Adjusted risk ratios were computed using Poisson regression with robust error variances. The analysis was adjusted for *a priori* confounders (age and sex) and variables associated with the outcome in the univariable analysis at a level of significance of p <0.2. The exposure of interest was effective treatment. The outcomes were bacteriological cure and a favourable clinical outcome (resolution or improvement) on follow up. Ethical approval for the ARGUS study was obtained from the Medical Research Council Zimbabwe (MRCZ/A/2406), the Institutional Review Board of the Biomedical Research and Training Institute in Zimbabwe and the London School of Hygiene and Tropical Medicine Ethics committee (Ref. 16424). The study was conducted in accordance with the Declaration of Helsinki and national and institutional standards. All participants provided written informed consent for participation in the study.

## Results

**Figure 1.**
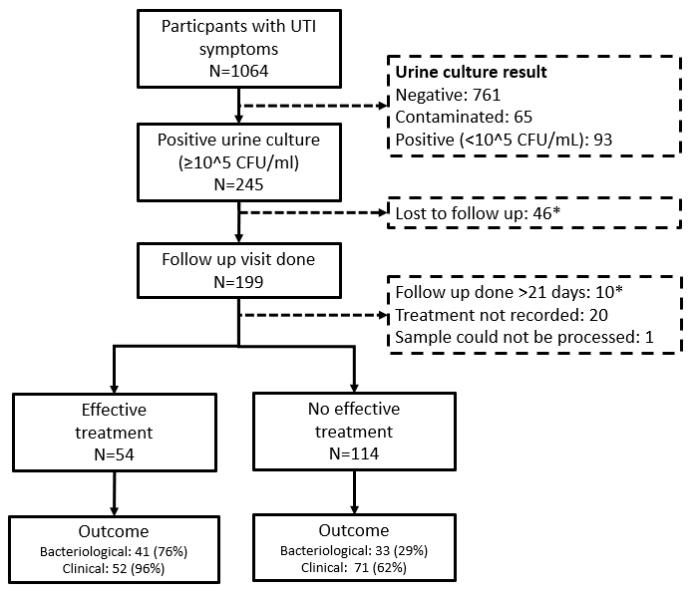
Flow chart of enrolled participants.

Of 1164 participants enrolled into the study, 245/1164 (21.0%) had a positive urine culture with ≥10 ^5^ colony forming units/mL at baseline and in 199/245 (81.2%), a follow up visit was conducted ( Figure 1) ^8^. This analysis excluded 31 participants. The reasons for exclusion are shown in Figure 1. The analysis included 168 participants with a median age of 33.6 years (IQR 25.1-51.4), of whom 131/168 (78.0%) were female. Participant characteristics are shown in Table 1. Effective treatment was taken by 54/168 (32.1%) participants, while in 61/168 (36.3%), treatment was ineffective due to resistance, in 35/168 (20.8%) no treatment was prescribed and 18/168 (10.7%) did not take the prescribed treatment. Antibiotic prescriptions were for amoxicillin in 59/168 (35.1%), fluoroquinolones in 51/168 (30.4%) and for other antibiotics in 15/168 (8.9%). Treatment was effective in 13/54 (24%) patients who took amoxicillin and in 41/46 (89%) patients who took a fluoroquinolone.

**Table 1.** Characteristics of study participants according to treatment status.

Characteristic	Total N=168	Effective treatment N=54	No effective treatment N=114	p-value
Age, median (IQR)	33.6 (25.1-51.4)	30.9 (24.2-52.0)	36.6 (25.7-49.5)	0.273
Female sex	131 (78.0)	43 (79.6)	88 (77.2)	0.722
HIV positive	50 (33.8))	9 (18.4)	41 (41.4)	0.005
Education (at least secondary)	129 (76.8)	40 (74.1)	89 (78.1)	0.567
Pregnancy	13 (10.6)	2 (5.3)	11 (12.9)	0.201
Time since the baseline visit	8 (7-11)	8 (7-10)	8 (7-11)	0.203
Duration of symptoms	6 (3-10)	5 (3-7)	7 (4-10)	0.032
Organism isolated (baseline) *E. coli* Coliforms *Enterococcus spp.* Other	137 (81.6) 21 (12.5) 8 (4.8) 2 (1.2)	44 (81.5) 6 (11.1) 3 (5.6) 1 (1.9)	93 (81.6) 15 (13.2) 5 (4.4) 1 (0.9)	-
Symptoms at day 7 Resolved completely Partial resolution Same or worse	59 (35.1) 64 (38.1) 45 (26.8)	31 (57.4) 21 (38.9) 2 (0.4)	28 (24.6) 43 (37.7) 43 (37.7)	<0.001

IQR: interquartile range; 20 participants did not know/disclose their HIV status; eight women did not know if they were pregnant.

The urine culture was negative at follow up in 41/54 (75.9%) of participants who took appropriate treatment and in 33/114 (28.9%, p<0.001) of those who did not. Symptoms had improved or resolved in 52/54 (96.3%) of those on appropriate treatment and in 71/114 (62.3%, p<0.001) of those without. Similarly, symptoms had improved or resolved in 67/74 (90.5%) of participants with a negative culture and in 56/94 (59.6%, p <0.001) of those with a positive culture. Participants who took effective treatment were more likely to have favourable clinical and bacteriological outcomes, with adjusted risk ratios of 1.42 (1.20-1.67) and 2.63 (1.77-3.92), respectively, when adjusting for age, sex, HIV status and duration of symptoms ( Table 2).

**Table 2.** Association between antibiotic treatment effectiveness and clinical and bacteriological outcomes.

Characteristic	RR (95%CI)	p-value	aRR (95%CI)	p-value
Clinical outcome				
Age (years)	0.99 (0.99-1.00)	0.890	0.99 (0.99-1.00)	0.660
Female sex	0.80 (0.68-0.95)	0.012	0.79 (0.64-0.97)	0.023
HIV infected	0.64 (0.48-0.85)	0.002	0.76 (0.59-0.99)	0.048
Duration of symptoms (days)	0.96 0.93-0.98)	0.001	0.97 (0.94-0.99)	0.014
Received effective treatment	1.55 (1.32-1.80)	<0.001	1.42 (1.20-1.67)	<0.001
Bacteriological outcome				
Age (years)	0.99 (0.99-1.01)	0.931	1.00 (0.99-1.01)	0.632
Female sex	0.82 (0.56-1.19)	0.291	0.76 (0.51-1.14)	0.188
HIV infected	0.64 (0.40-1.03)	0.064	0.87 (0.55-1.39)	0.568
Duration of symptoms (days)	0.93 (0.89-0.98)	0.006	0.96 (0.92-1.01)	0.154
Received effective treatment	2.62 (1.89-3.63)	<0.001	2.63 (1.77-3.92)	<0.001

*RR: risk ratio; aRR: adjusted RR; CI: confidence interval; age and duration of symptoms were analysed as continuous variables; duration of symptoms refers to the duration of symptoms prior to the initial clinic presentation (baseline visit).*

## Discussion

This study shows that individuals who receive treatment against which the pathogen is sensitive have a significantly higher chance of experiencing clinical and bacteriological cure on follow up than those given ineffective treatment or not treated at all.

In line with other studies symptom resolution or improvement was common even in those participants who did not take effective treatment. Spontaneous symptom resolution of uncomplicated cystitis occurs in about a third of patients ^9^. However, patients without spontaneous cure often experience debilitating symptoms that can persist for several weeks ^10^, affecting quality of life ^11^ and leading to economic costs due to loss of income, missed days at work and medical expenses ^1, 12, 13^. Effective empiric treatment increases the speed and likelihood of symptom resolution and therefore is an important intervention at primary care level.

More than half of participants who took the prescribed antibiotics had infections with organisms that were resistant to the antibiotic prescribed. Resistance to amoxicillin was more frequent (76%) compared to fluoroquinolones (11%), which are recommended as the first-line empiric treatment of UTIs in outpatients in Zimbabwe ^7^. The high prevalence of amoxicillin resistance is not surprising and is in line with other reports from sub-Saharan Africa ^14^. In view of the high prevalence of amoxicillin resistance, recommended use of the drug for empiric treatment of UTI needs to be reviewed. Although fluoroquinolones are safe and resistance is less frequent, they may not be the optimal choice as first-line drugs due to their potential for resistance development ^15^, particularly when other low-cost alternatives such as nitrofurantoin and fosfomycin are available. Furthermore, fluoroquinolone resistance is increasing in many settings ^16^, likely driven by selective pressures exerted by their use and the dissemination of successful bacterial clones ^17^. This highlights the need to optimise treatment recommendations based on setting-specific AST data.

In this study, effective treatment according to AST resulted in more frequent bacteriological eradication of infection and improved clinical outcomes. While effective treatment has been shown to be associated with symptom resolution and bacteriological clearance in high-income settings ^18– 20^, similar data from sub-Saharan Africa are scarce. Some participants did not fill their antibiotic prescription, potentially due to costs. In addition, while healthcare workers may be aware that amoxicillin is not very effective, it may have been the only inexpensive antibiotic available. Thus, costs and stock outs may impact on antibiotic prescriptions and consequently on the effectiveness of treatment.

The study is limited by the relatively high rate of loss to follow up explained by the economic hardships in Zimbabwe, preventing participants from travelling to the clinics for their follow up visits, and the ongoing COVID-19 pandemic, which led to clinic closures and a national lockdown, preventing successful follow-up. An important strength of this study was the follow up of participants using culture to determine the effect of treatment on both clinical symptoms and bacteriological cure. In this study, a high proportion of participants did not receive effective treatment which may not be the case in other settings where antibiotics recommended for UTI treatment reflect the local prevalence of AMR and where effective antibiotics are more available and affordable. However, the impact of effective treatment on patient outcomes would not be different between settings making these results generalizable.

The findings of this study show that there is a high proportion of untreated UTIs, with patients often being prescribed antibiotics that are ineffective or not being able to afford treatment.

Furthermore, the long duration of symptoms prior to presentation suggests that some patients with UTIs may not present to clinics at all. Optimising treatment recommendations could be achieved by conducting sentinel-site surveillance for determining the prevalence of resistance, which would in turn inform empiric treatment. While UTIs have not received a lot of attention, partly because a high proportion resolve without treatment and partly because they are not life-threatening and the long-term morbidity is limited, they do impact on quality of life, particularly in women, and lead to loss of income and economic costs.

## Data availability

### Underlying data

Dryad: ARGUS clinical and bacteriological outcomes dataset. https://doi.org/10.5061/dryad.v41ns1rwb ^8^.

This project contains the following underlying data:

- ARGUS_outcome_data.csv
- ARGUS_outcome_codebook.docx

### Reporting guidelines

Dryad: STROBE checklist for “Clinical and bacteriological outcomes in patients with urinary tract infections presenting to primary care in Harare, Zimbabwe: a cohort study”. https://doi.org/10.5061/dryad.v41ns1rwb ^8^.

Data are available under the terms of the Creative Commons Zero "No rights reserved" data waiver (CC0 1.0 Public domain dedication).
