# Supplementary material for: Clinical and bacteriological outcomes in patients with urinary tract infections presenting to primary care in Harare, Zimbabwe: a cohort study
Source: Wellcome Open Res. 2022 Aug 17;6:135. Originally published 2021 Jun 1. [Version 2] doi: 10.12688/wellcomeopenres.16789.2 (PMC9403354; doi:10.12688/wellcomeopenres.16789.2)
Supplement: Peer review history

# Peer review: Reviewer response for version 2

**DOI:** 10.21956/wellcomeopenres.20240.r52049

**Published:** 2022-09-05

**Authors:** Sören L. Becker

**Affiliations:** 1 Institute of Medical Microbiology and Hygiene, Saarland University, Homburg, Germany

The authors have adequately addressed all my concerns and the paper can be indexed in its revised form.

Is the work clearly and accurately presented and does it cite the current literature?

Partly

If applicable, is the statistical analysis and its interpretation appropriate?

Yes

Are all the source data underlying the results available to ensure full reproducibility?

Partly

Is the study design appropriate and is the work technically sound?

Yes

Are the conclusions drawn adequately supported by the results?

Yes

Are sufficient details of methods and analysis provided to allow replication by others?

Partly

Reviewer Expertise:

NA

I confirm that I have read this submission and believe that I have an appropriate level of expertise to confirm that it is of an acceptable scientific standard.

# Peer review: Reviewer response for version 2

**DOI:** 10.21956/wellcomeopenres.20240.r52048

**Published:** 2022-08-24

**Authors:** Niels Frimodt-Møller

**Affiliations:** 1 Department of Clinical Microbiology, Rigshospitalet, University of Copenhagen, Copenhagen, Denmark

I have no further objections – the authors seem to have reacted appropriately to the comments from the reviewers.

Is the work clearly and accurately presented and does it cite the current literature?

Yes

If applicable, is the statistical analysis and its interpretation appropriate?

Yes

Are all the source data underlying the results available to ensure full reproducibility?

Yes

Is the study design appropriate and is the work technically sound?

Yes

Are the conclusions drawn adequately supported by the results?

Yes

Are sufficient details of methods and analysis provided to allow replication by others?

No

Reviewer Expertise:

Specialist in clinical microbiology with >40 years of research interest in UTI, and have performed research in West Africa on AMR

I confirm that I have read this submission and believe that I have an appropriate level of expertise to confirm that it is of an acceptable scientific standard.

# Peer review: Reviewer response for version 1

**DOI:** 10.21956/wellcomeopenres.18519.r51675

**Published:** 2022-08-10

**Authors:** Sören L. Becker

**Affiliations:** 1 Institute of Medical Microbiology and Hygiene, Saarland University, Homburg, Germany

This is an interesting study on the aetiology and treatment outcome of adult individuals with community-onset urinary tract infections in Zimbabwe. There is a paucity of data on this topic from sub-Saharan Africa, and the article thus provides interesting information. However, I would recommend that the following recommendations should be addressed:

 1) Please give details on which laboratory performed the microbiological examination. How was the sample transfer organised? Also, please give some information on the employed agar media and the incubation period. Which tests were used to distinguish Escherichia coli from other coliform bacteria and Gram-positive pathogens?

 2) Please detail for how many days and at which dosage the empirical treatment with amoxicillin or a fluoroquinolone (which one was used?) was employed.

 3) In addition to the names of the identified bacterial pathogens, please also provide data -- at least in the text -- on the antimicrobial susceptibility patterns. In the Discussion, you mention fosfomycin and nitrofurantoin as potential alternative treatment options. Did you also assess their susceptibility pattern?

 4) It might be worthwhile to mention that some urinary tract infections may also give rise to severe consequences if left untreated, e.g. pyelonephritis and/or bacteraemia.

 5) There are some recent manuscripts from neighbouring countries in sub-Saharan Africa on similar topics, which might also be mentioned in the Discussion.

Is the work clearly and accurately presented and does it cite the current literature?

Partly

If applicable, is the statistical analysis and its interpretation appropriate?

Yes

Are all the source data underlying the results available to ensure full reproducibility?

Partly

Is the study design appropriate and is the work technically sound?

Yes

Are the conclusions drawn adequately supported by the results?

Yes

Are sufficient details of methods and analysis provided to allow replication by others?

Partly

Reviewer Expertise:

Epidemiology, Antimicrobial resistance, Clinical microbiology

I confirm that I have read this submission and believe that I have an appropriate level of expertise to confirm that it is of an acceptable scientific standard, however I have significant reservations, as outlined above.

# Response

**Published:** 2022-08-14

**Authors:** Ioana Diana Olaru

**Affiliations:** London School of Hygiene & Tropical Medicine, UK

Thank you for your comments on the submitted manuscript. Please find the responses to the comments below. Changes reflecting the comments have been made to the manuscript according to your suggestions.

**Comment #1**

 Please give details on which laboratory performed the microbiological examination. How was the sample transfer organised? Also, please give some information on the employed agar media and the incubation period. Which tests were used to distinguish Escherichia coli from other coliform bacteria and Gram-positive pathogens?

**Response to comment #1**

 Urine cultures were performed at the Biomedical Research and Training Institute Laboratory which is a research laboratory with expertise in microbiology. Samples were transported to the laboratory on the same day of collection by a dedicated driver. Urine samples were inoculated on chromogenic agar (Brilliance UTI agar, Oxoid, UK) and incubated for 24h at 37°C. Bacterial identification was performed using APIs (bioMerieux, France) and conventional microbiological techniques (catalase, coagulase). *E. coli* isolates also underwent whole genome sequencing. A more detailed description of microbiological procedures and antimicrobial testing results for this patient cohort was already published (Olaru ID et al. JAC-AMR, 2021). Clarifications on microbiology procedures were added to the manuscript.

*“Samples were processed on the same day at the Biomedical Research and Training Institute Laboratory and were delivered to the laboratory within 6 hours of collection.”*

*“Urine samples were inoculated on chromogenic agar (Brilliance UTI agar, Oxoid, UK) and incubated for 24 hours at 37°C. Bacterial identification was performed using biochemical tests (API 20E, bioMérieux, France, catalase, coagulase).”*

**Comment #2**

 Please detail for how many days and at which dosage the empirical treatment with amoxicillin or a fluoroquinolone (which one was used?) was employed.

**Response to comment #2**

 Patients with suspected UTIs were treated empirically according to the national Zimbabwean guidelines by the healthcare workers providing routine care in the clinics. For cystitis, the guidelines recommend amoxicillin for 500mg tid for 3 days or a fluoroquinolone, usually ciprofloxacin 500 mg bd. For pyelonephritis treatment is recommended for 2 weeks and patients with more severe disease are referred to hospital for intravenous treatment with ceftriaxone or gentamicin.

 A clarification was added to the manuscript

*“Treatment was prescribed by the clinic nurses according to routine practice and followed the national guidelines, which recommend amoxicillin 500 mg thrice daily or fluoroquinolones, usually ciprofloxacin 500 mg twice daily, for 3 days as first-line treatment for cystitis”*

**Comment #3**

 In addition to the names of the identified bacterial pathogens, please also provide data -- at least in the text -- on the antimicrobial susceptibility patterns. In the Discussion, you mention fosfomycin and nitrofurantoin as potential alternative treatment options. Did you also assess their susceptibility pattern?

**Response to comment #3**

 Information on susceptibility patterns was added to the manuscript and is described more extensively in Olaru ID et al. JAC-AMR, 2021. Susceptibility testing was also performed for nitrofurantoin and fosfomycin. Among *E. coli,* fosfomycin resistance was present in 3/137 (2.2%) however unlike nitrofurantoin, fosfomycin is not readily available in Zimbabwe.

*“Among the 158 Enterobacterales isolates identified, the prevalence of resistance was 137/158 (86.7%) for amoxicillin, 132/158 (83.4%) for trimethoprim-sulfamethoxazole, 27/158 (17.1%) for ciprofloxacin, 23/158 (14.6%) for ceftriaxone, and 16/158 (10.1%) for nitrofurantoin.”*

**Comment #4**

 It might be worthwhile to mention that some urinary tract infections may also give rise to severe consequences if left untreated, e.g. pyelonephritis and/or bacteraemia.

**Response to comment #4**

 A comment was added: *“In the absence of effective treatment, some lower UTIs may also progress to pyelonephritis and bacteraemia which may in turn lead to hospitalisation and severe outcomes.”*

**Comment #5**

 There are some recent manuscripts from neighbouring countries in sub-Saharan Africa on similar topics, which might also be mentioned in the Discussion.

**Response to comment #5**

 Thank you for your comment. The findings of this study were put into context with other studies from sub-Saharan Africa which were added to the discussion: *“The high prevalence of amoxicillin resistance is not surprising and is in line with reports from other countries in sub-Saharan Africa ^14,24,25^.”*

*References*

*14. Tadesse BT, Ashley EA, Ongarello S, et al.: Antimicrobial resistance in Africa: a systematic review. BMC Infect Dis. 2017;17(1):616. 28893183 10.1186/s12879-017-2713-1 5594539*

*24. Schmider J, Bühler N, Mkwatta H, et al.* *Microbiological Characterisation of Community-Acquired Urinary Tract Infections in Bagamoyo, Tanzania: A Prospective Study. Trop Med Infect Dis. 2022 Jun 12;7(6):100. doi: 10.3390/tropicalmed7060100.*

*25. Abongomera G, Koller M, Musaazi J, et al. Spectrum of antibiotic resistance in UTI caused by Escherichia coli among HIV-infected patients in Uganda: a cross-sectional study. BMC Infect Dis. 2021 Nov 23;21(1):1179. doi: 10.1186/s12879-021-06865-3.*

# Peer review: Reviewer response for version 1

**DOI:** 10.21956/wellcomeopenres.18519.r51679

**Published:** 2022-08-05

**Authors:** Lynora Saxinger

**Affiliations:** 1 Division of Infectious Diseases, Department of Medicine, University of Alberta, Edmonton, AB, Canada

This study essentially describes the adequacy of outpatient empiric therapy for uncomplicated UTI in Zimbabwe. This is clinically relevant inasmuch as it highlights the need for reassessment of guidelines in light of geographically assessed antimicrobial resistance patterns.

 Suggest comment on:

1. Only 1/5 of patients who had 2 UTI symptoms were culture positive: were competing clinical diagnoses assessed? (urethritis-vulvovaginitis) or possible culture issues.
2. Reason for 20% of clinical UTI patients not receiving antibiotics is not discussed.
3. Results: describe overall resistance profile by organism.
4. Don't need as much detail describing association of adequate therapy with microbiologic and clinical resolution, I'd expand resistance stewardship considerations (which are appropriate) instead.  

Is the work clearly and accurately presented and does it cite the current literature?

Partly

If applicable, is the statistical analysis and its interpretation appropriate?

Yes

Are all the source data underlying the results available to ensure full reproducibility?

Yes

Is the study design appropriate and is the work technically sound?

Yes

Are the conclusions drawn adequately supported by the results?

Yes

Are sufficient details of methods and analysis provided to allow replication by others?

Yes

Reviewer Expertise:

Infectious diseases, antimicrobial stewardship and resistance, guidelines and knowledge translation.

I confirm that I have read this submission and believe that I have an appropriate level of expertise to confirm that it is of an acceptable scientific standard, however I have significant reservations, as outlined above.

# Response

**Published:** 2022-08-14

**Authors:** Ioana Diana Olaru

**Affiliations:** London School of Hygiene & Tropical Medicine, UK

Thank you for your comments on the submitted manuscript. Please find the responses to the comments below. Changes reflecting the comments have been made to the manuscript according to your suggestions.

**Comment #1**

 Only 1/5 of patients who had 2 UTI symptoms were culture positive: were competing clinical diagnoses assessed? (urethritis-vulvovaginitis) or possible culture issues.

**Response to comment #1**

 The yield of urine cultures in this study was much lower than in high-income settings where >80% of patients with acute urinary tract symptoms have a positive urine culture. A similarly low yield of urine cultures was observed in other studies from sub-Saharan Africa (e.g. 36% in the study by Iregbu et al. BMC Infect Dis 2017). In separate analyses, we explored possible causes of the low yield. We found (Olaru ID et al. BMJ Open 2021) that almost 20% of patients had a sexually transmitted infection and 20% had antibiotic exposure prior to seeking care. A laboratory issue which may result in a low yield is unlikely as we also evaluated the performance of alternative culture media inoculated at the point of care (Olaru ID et al. Eur J Clin Microbiol Infect Dis, 2021). We did not observe a lower yield in samples inoculated in the laboratory, and results of the different methods were mostly concordant. A comment addressing these issues was added to the manuscript.

*“The yield of urine cultures was low in line with findings from other studies from sub-Saharan Africa. This can be explained in part by the high prevalence of sexually transmitted infections and antibiotic exposure prior to seeking healthcare among patients included in this study.”*

**Comment #2**

 Reason for 20% of clinical UTI patients not receiving antibiotics is not discussed.

**Response to comment #2**

 The decision to prescribe antibiotics was made by the healthcare providers working at the clinic and not by the research staff. Patients with positive urine cultures were followed up by the research staff and if no effective antibiotics had been prescribed and symptoms had not resolved (or they were pregnant) they were issued antibiotics according to the susceptibility testing results. Healthcare workers may have suspected an alternative diagnosis in the 20% of patients who were not prescribed antibiotics at the initial consultation. A comment was added in the manuscript discussion:

*“Healthcare workers did not prescribe antibiotics at the initial consultation in one out of five patients with a positive urine culture. This may be due to uncertainties on the part of the healthcare workers that the patient has an UTI and to suspected alternative diagnoses.”*

**Comment #3**

 Results: describe overall resistance profile by organism.

**Response to comment #3**

 A sentence on the prevalence of resistance was added to the manuscript: *“Among the 158 Enterobacterales isolates identified, the prevalence of resistance was 137/158 (86.7%) for amoxicillin, 132/158 (83.4%) for trimethoprim-sulfamethoxazole, 27/158 (17.1%) for ciprofloxacin, 23/158 (14.6%) for ceftriaxone, and 16/158 (10.1%) for nitrofurantoin.” *The prevalence of resistance according to bacterial species is discussed in more detail in a separate analysis of the baseline population. (Olaru ID et al. JAC-AMR, 2021)

**Comment #4**

 Don't need as much detail describing association of adequate therapy with microbiologic and clinical resolution, I'd expand resistance stewardship considerations (which are appropriate) instead.  

**Response to comment #4**

 The focus of the current manuscript is on the clinical and bacteriological outcomes of patients with culture-confirmed urinary tract infections. Although the importance of effective treatment is widely recognised, few studies from sub-Saharan Africa discuss this issue and treatment guidelines in this setting often do not reflect the local epidemiology of resistance. We fully agree that antibiotic stewardship is critical in preventing the further spread of AMR. This has been further discussed in the manuscript:

*“Limited availability of diagnostics for sexually transmitted infections does not allow the differentiation between UTIs and sexually transmitted infections. Also, the lack of up to date AMR data can lead to excessive and inappropriate antibiotic use which will further drive the development of AMR. These in turn can only be addressed by sustained investment in laboratory strengthening for obtaining locally-representative surveillance data, and in improving access to diagnostics and to healthcare in general.”*

# Peer review: Reviewer response for version 1

**DOI:** 10.21956/wellcomeopenres.18519.r44213

**Published:** 2021-06-16

**Authors:** Niels Frimodt-Møller

**Affiliations:** 1 Department of Clinical Microbiology, Rigshospitalet, University of Copenhagen, Copenhagen, Denmark

The paper presents the results of a study on the diagnosis and treatment of urinary tract infections in a low-income setting, which makes it an important contribution to our understanding of the consequences of interventions in that type of setting. The report is well written, and the study setup fine.

1. It is always important for the understanding of the results to provide details on urine sampling - mid stream voided urine? Cleaning of meatus urethrae before sampling? Time from sampling to culture?
2. The study provides important results on the feasibility of the standard treatment and shows, perhaps not surprisingly, that amoxicillin is not a perfect choice for empiric treatment in that setting. Also, the authors are correct in discussing that ciprofloxacin should be reserved for hospital treatment and not used on a broad scale. I miss a discussion on the finding that 761 patients had negative culture and were treated with antibiotics anyway - if I understand correctly. This is perhaps more worrisome in view of the large misuse of anitibotics; how would the authors improve diagnostics to avoid such mishaps?
3. Some data on antibiotic resistance in the pathogens found would be interesting.

Is the work clearly and accurately presented and does it cite the current literature?

Yes

If applicable, is the statistical analysis and its interpretation appropriate?

Yes

Are all the source data underlying the results available to ensure full reproducibility?

Yes

Is the study design appropriate and is the work technically sound?

Yes

Are the conclusions drawn adequately supported by the results?

Yes

Are sufficient details of methods and analysis provided to allow replication by others?

No

Reviewer Expertise:

Specialist in clinical microbiology with >40 years of research interest in UTI, and have performed research in West Africa on AMR

I confirm that I have read this submission and believe that I have an appropriate level of expertise to confirm that it is of an acceptable scientific standard, however I have significant reservations, as outlined above.

# Response

**Published:** 2022-08-14

**Authors:** Ioana Diana Olaru

**Affiliations:** London School of Hygiene & Tropical Medicine, UK

**Comment #1**

 It is always important for the understanding of the results to provide details on urine sampling - mid stream voided urine? Cleaning of meatus urethrae before sampling? Time from sampling to culture?

**Response to comment #1**

 Thank you for your comments. Clarifications were added in the methods section:

*“After cleaning the urethral meatus and the perineal region, a mid-stream urine sample was collected for culture.”*

*“Samples were processed on the same day at the Biomedical Research and Training Institute Laboratory and were transported to the laboratory at 4-8°C within 6 hours of collection.”*

**Comment #2**

 The study provides important results on the feasibility of the standard treatment and shows, perhaps not surprisingly, that amoxicillin is not a perfect choice for empiric treatment in that setting. Also, the authors are correct in discussing that ciprofloxacin should be reserved for hospital treatment and not used on a broad scale. I miss a discussion on the finding that 761 patients had negative culture and were treated with antibiotics anyway - if I understand correctly. This is perhaps more worrisome in view of the large misuse of antibiotics; how would the authors improve diagnostics to avoid such mishaps?

**Response to comment #2**

 We fully agree with the reviewer that overprescribing of antibiotics is a critical issue in low- and middle-income settings where diagnostics for infections are usually not available.

 Of the 761 patients with a negative urine culture, 55% were treated with antibiotics including treatment for sexually transmitted infections. In a separate analysis of a subset of patients from the same cohort (Olaru ID et al. BMJ Open 2021) we explored possible causes of negative urine cultures. We found that almost 20% of patients (the majority of whom had negative urine cultures) had a sexually transmitted infection. Also 20% of patients with urinary symptoms had antibiotic exposure in the community prior to accessing healthcare. Therefore, some patients may have had a partially treated urinary tract infection resulting in negative cultures. A statement was added on improving diagnostics as suggested:

*“The yield of urine cultures was low in line with findings from other studies from sub-Saharan Africa ^21,22^. This can be explained in part by the high prevalence of sexually transmitted infections and antibiotic exposure prior to seeking healthcare among patients included in this study ^23^. The availability of rapid diagnostic tests at the point of care such as urinary dipstick or testing for sexually transmitted infections may improve patient care and optimise antibiotic use.”*

*References: 21 Iregbu KC, Nwajiobi-Princewill PI. Urinary tract infections in a tertiary hospital in Abuja, Nigeria. African Journal of Clinical and Experimental Microbiology 2013;14:169–73.*

*22 Bitew A, Molalign T, Chanie M. Species distribution and antibiotic susceptibility profile of bacterial uropathogens among patients complaining urinary tract infections. BMC Infect Dis 2017;17:654.*

*23 Olaru ID, Chisenga M, Yeung S, et al.* *Sexually transmitted infections and prior antibiotic use as important causes for negative urine cultures among adults presenting with urinary tract infection symptoms to primary care clinics in Zimbabwe: a cross-sectional study.* *BMJ Open. 2021 Aug 11;11(8):e050407. doi: 10.1136/bmjopen-2021-050407.*

**Comment #3**

 Some data on antibiotic resistance in the pathogens found would be interesting.

**Response to comment #3**

 A sentence on the prevalence of resistance was added to the manuscript: *“Among the 158 Enterobacterales isolates identified, the prevalence of resistance was 137/158 (86.7%) for amoxicillin, 132/158 (83.4%) for trimethoprim-sulfamethoxazole, 27/158 (17.1%) for ciprofloxacin, 23/158 (14.6%) for ceftriaxone, and 16/158 (10.1%) for nitrofurantoin.” *The prevalence of resistance according to bacterial species is discussed in more detail in a separate analysis of the baseline population. (Olaru ID et al. JAC-AMR, 2021)
